# Identification of Novel Contributions to High-affinity Glycoprotein–Receptor Interactions using Engineered Ligands

**DOI:** 10.1016/j.jmb.2009.11.073

**Published:** 2010-02-26

**Authors:** Peter J. Coombs, Rebecca Harrison, Samantha Pemberton, Adrián Quintero-Martinez, Simon Parry, Stuart M. Haslam, Anne Dell, Maureen E. Taylor, Kurt Drickamer

**Affiliations:** Division of Molecular Biosciences, Department of Life Sciences, Imperial College, London SW7 2AZ, UK

**Keywords:** CRD, carbohydrate-recognition domain, ECD, extracellular domain, DC-SIGN, dendritic cell-specific intercellular adhesion molecule 3-grabbing nonintegrin, MGL, macrophage galactose lectin, RHL-1, rat hepatic lectin 1, OR, orosomucoid, SBA, soybean agglutinin, glycan-binding receptors, lectins, glycoproteins, affinity

## Abstract

Engineered receptor fragments and glycoprotein ligands employed in different assay formats have been used to dissect the basis for the dramatic enhancement of binding of two model membrane receptors, dendritic cell-specific intercellular adhesion molecule 3-grabbing nonintegrin (DC-SIGN) and the macrophage galactose lectin, to glycoprotein ligands compared to simple sugars. These approaches make it possible to quantify the importance of two major factors that combine to enhance the affinity of single carbohydrate-recognition domains (CRDs) for glycoprotein ligands by 100-to 300-fold. First, the presence of extended binding sites within a single CRD can enhance interaction with branched glycans, resulting in increases of fivefold to 20-fold in affinity. Second, presentation of glycans on a glycoprotein surface increases affinity by 15-to 20-fold, possibly due to low-specificity interactions with the surface of the protein or restriction in the conformation of the glycans. In contrast, when solution-phase networking is avoided, enhancement due to binding of multiple branches of a glycan to multiple CRDs in the oligomeric forms of these receptors is minimal and binding of a receptor oligomer to multiple glycans on a single glycoprotein makes only a twofold contribution to overall affinity. Thus, in these cases, multivalent interactions of individual glycoproteins with individual receptor oligomers have a limited role in achieving high affinity. These findings, combined with considerations of membrane receptor geometry, are consistent with the idea that further enhancement of the binding to multivalent glycoprotein ligands requires interaction of multiple receptor oligomers with the ligands.

## Introduction

**Fig. 1:**
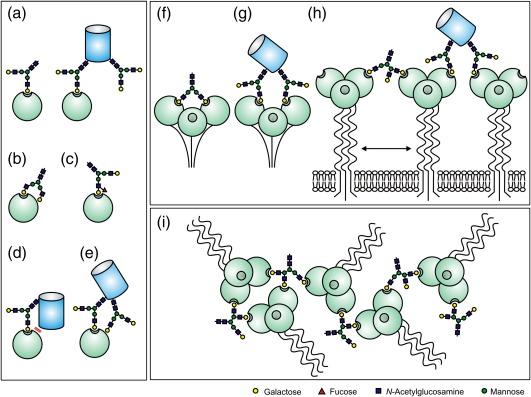
Potential sources of enhanced affinities in glycoprotein-receptor interactions. (a) Glycan branching and attachment of multiple glycans to a glycoprotein increases the number of terminal sugar residues. (b) and (c) Extended binding site interactions can accommodate secondary contact with branches on multi-antennary glycans or with terminal elaborations on individual branches. (d) Direct protein–protein interactions can occur between a CRD and the surface of the protein portion of the glycoprotein. (e) The presence of multiple glycans on a glycoprotein ligand can lead to secondary interactions. (f) and (g), Multiple terminal residues on one glycan or on different glycans attached to a glycoprotein can interact with multiple CRDs in a receptor oligomer. (h) On cell surfaces, CRDs in receptor oligomers can interact with glycans on multiple glycoprotein ligands. (i) In solution, lattices can form from interactions of multivalent ligands with oligomeric lectins. CRDs are shown diagrammatically as spheres and glycoprotein ligands are indicated as cylinders. A galactose-binding receptor is shown for illustration purposes.

Receptor-mediated binding of sugars underpins recognition events important for cell–cell adhesion, serum glycoprotein clearance, innate immune recognition of pathogens and other biological processes.^1-4^ Such glycan-mediated binding events in solution and at cell surfaces typically involve multimeric glycan-binding receptors, also known as lectins, interacting with glycoproteins bearing multiple oligosaccharides. The importance of multivalency in the interaction of glycans with receptors is widely documented for glycan-binding molecules of diverse bacterial, plant and animal origin.^5-8^ Levels of enhancement of ligand binding resulting from multivalency have reached 10^6^-fold in some assay conditions.^5,9,10^ For the C-type animal lectins, a diverse group of glycan-binding receptors containing carbohydrate-recognition domains (CRDs) that share a Ca^2+^-dependent mechanism of interaction with monosaccharide residues, the fundamental binding interaction is typically very weak, with dissociation constants of the order of 1 mM.^11-13^ A number of different factors have been invoked to explain how affinity and specificity of glycan-binding receptors containing C-type CRDs can be increased to achieve biologically relevant interactions (Fig. 1). In spite of the high levels of affinity enhancement reported for earlier studies, the contributions of these individual factors have often been difficult to dissect and some affinity enhancements have been measured under conditions that would not be relevant to the natural biological situation. For example, the three-dimensional network arrangement seen for soluble lectins^14^ (Fig. 1i) would not generally be possible for membrane-resident glycan-binding receptors.

New experimental approaches to measuring binding interactions have facilitated exploration of some of the sources of affinity in greater detail. For example, the importance of ligand mobility for interaction with multiple CRDs has been studied recently using surface-force measurements of the receptor dendritic cell cell-specific intercellular adhesion molecule 3-grabbing nonintegrin (DC-SIGN), which is a tetramer that binds to high-mannose oligosaccharides such as those found on viral pathogens, including human immunodeficiency virus.^13,15,16^ The results demonstrate that conformational flexibility in the dispositions of CRDs in this tetrameric receptor observed in structural analysis^17,18^ makes interactions of a single oligomer with multiple glycans possible. Such interactions, combined with ligand mobility, would facilitate two-dimensional lattice formation (Fig. 1h). A further recent development is the ability to engineer both receptors and their glycoprotein ligands. In the case of DC-SIGN, the ability to express isolated monomeric CRDs and tetrameric extracellular domains of the receptor allows comparison of the effects of CRD clusters.^13^ Using this approach, it has been possible to demonstrate that the CRD of DC-SIGN shows tenfold higher affinity for a branched glycan than for mannose, demonstrating the importance of interactions of a single CRD with multiple branches of a single glycan (Fig 1b). Structural analysis has shown that this enhanced affinity results from an extended binding site on the surface of the CRD, allowing interaction with the branched oligosaccharide structure.^19^ The possibility of producing engineered glycoproteins as ligands for receptors has been less exploited to date. Also, very few measurements of single glycan–CRD interactions have been reported, in part because these do not fall into an affinity range that is easily assayed, but label-free methods such as surface plasmon resonance have the potential to address this gap.

In the current studies, the behavior of isolated CRDs and CRDs clustered in naturally occurring oligomers have been compared in parallel conditions, using engineered glycoprotein ligands presenting different numbers of glycans on a common backbone in multiple assay formats. The results using DC-SIGN and the structurally and functionally divergent macrophage galactose lectin as model receptors reveal that affinity enhancements for binding of glycoprotein ligands of greater than 1000-fold can result from the combined effects of presentation on a protein surface and the presence of extended binding sites in the receptor CRDs.

## Results

### Using DC-SIGN to assess affinity enhancement resulting from glycan presentation on proteins

**Fig. 2:**
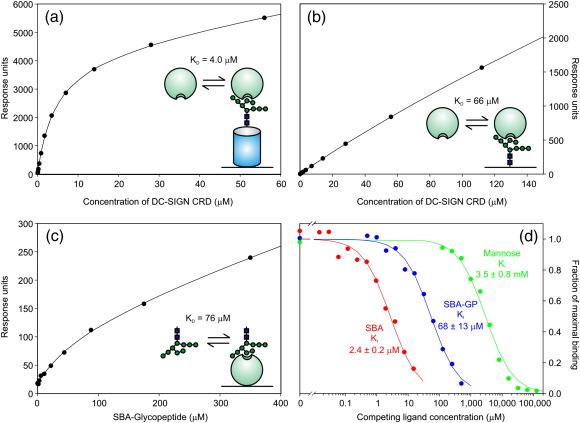
Determination of the affinity of DC-SIGN for glycopeptide and glycoprotein ligands. (a) and (b) Binding of monomeric CRD to immobilized SBA and glycopeptide derived from SBA. Data were fit to simple first-order binding curves with a linear increase in nonspecific background binding to derive dissociation constants. (c) Binding of the glycopeptide from SBA to immobilized CRD from DC-SIGN. *K*_D_ was derived from fitting as in the previous experiments. (d) Binding competition assays in which immobilized CRD from DC-SIGN was probed with ^125^I-labeled mannose-BSA in the presence of competing ligands. Data were fit to simple first-order competition curves to derive *K*_I_ values.

In initial studies, DC-SIGN was used to demonstrate the importance of some potential factors in enhancing glycoprotein binding to a CRD and to validate appropriate assay formats. The availability of a highly soluble, monomeric CRD from DC-SIGN and the fact that high-mannose oligosaccharides bind to this receptor with affinities that fall within the range that can be determined using surface plasmon resonance provided an opportunity to measure the interaction directly. Making use of the fact that each subunit of soybean agglutinin (SBA) bears a single, uniform Man_9_GlcNAc_2_ oligosaccharide,^20^ N-hydroxysuccinimide chemistry was used to prepare surfaces coated with the intact glycoprotein and with the glycopeptide isolated from it, in order to assess the effect of glycan presentation on a protein backbone (Fig. 1d). Effects of valency were eliminated by analyzing the interaction with monomeric CRDs. Because of the rapid rates of association and dissociation, interactions were measured under steady-state conditions and analyzed by fitting the data to a simple saturation binding model (Fig. 2a and b). The results suggest that presentation of the oligosaccharide on a glycoprotein results in a roughly 15-fold enhancement of the binding affinity. However, the data for the glycopeptide binding experiment fall mostly below the apparent *K*_D_ obtained from fitting the curve, because of limits on the protein concentration that could be achieved. A more definitive result was obtained by immobilizing the CRD, because higher concentrations of glycopeptide could be achieved in the fluid phase (Fig. 2c). The measured *K*_D_ of 76 μM suggests that the enhancement resulting from presentation of the Man_9_GlcNAc_2_ oligosaccharide on a protein scaffold is closer to 20-fold.

The same interactions were compared in a binding competition assay, in which the CRD was immobilized on a polystyrene surface and competition for binding to an iodinated mannose–BSA reporter ligand was measured (Fig. 2d). These experiments were done with concentrations of reporter ligand at least tenfold below the *K*_D_ value for the reporter ligand, in a region where bound ligand is linearly proportional to input ligand concentration. Under such conditions, the *K*_I_ values for the competing ligands closely approximate their *K*_D_ values.^21^ The ratio of *K*_I,mannose_ to *K*_I,glycopeptide_ is 51 (3.5 mM/68 μM), which indicates that DC-SIGN binds to a Man_9_GlcNAc_2_ glycopeptide with more than 50-fold higher affinity than it binds to mannose. On the basis of the presence of three terminal mannose residues, the affinity for the glycopeptide would be expected to be only threefold higher than the affinity for mannose, so the actual affinity ratio is roughly 51/3, which is 17-fold higher than would be expected. This value is consistent with previous studies and can be explained on the basis of the enhanced affinity for the branched mannose structure that has been observed to interact with an extended binding site in the CRD.^22,23^ The ratio of the affinities for the glycopeptide and the intact soybean agglutinin (*K*_I,glycopeptide_/*K*_I,SBA_ =  68 μM/2.4 μM) suggests a further enhancement of 28-fold for binding to the glycoprotein compared to the glycopeptide.

The absolute values of the affinities measured in the competition assay and the enhancement resulting from presentation of the oligosaccharide on a protein scaffold are remarkably consistent with the values from the direct binding analysis, considering the widely different assay formats involved. This result suggests that the competition assay also measures largely a single glycan–CRD interaction. For the free glycopeptide, this is expected, but it might seem more surprising for the intact glycoprotein, which is tetrameric. However, the multiple glycans on the tetramer are apparently not well disposed for binding to the CRDs exposed on a surface. Only two glycans are presented on each face of the soybean agglutinin tetramer,^24^ and the close spacing of these glycans relative to the size of a CRD, as well as the precise orientation required to accommodate the oligosaccharide in the extended binding site on the CRD, apparently make it difficult to align more than one glycan for high-affinity binding to CRDs immobilized on a plastic surface and there is minimal enhancement in affinity. The consistency of the results in these different assay formats validates the competition assay as a way to compare affinities, which allows analysis of other receptor systems where the surface plasmon resonance approach is not applicable.

Comparing the affinity of the CRD from DC-SIGN for mannose as measured in the competition assay (*K*_I_ =  3.5 mM; Fig. 2d) with the most conservative value for the CRD–glycoprotein interaction that comes from the surface plasmon resonance experiment (*K*_D_ =  4.0 μM; Fig. 2a), the ratio of affinities is 875. This affinity is nearly 300-fold higher than the threefold enhancement that would be expected based on the presence of three terminal mannose residues per soybean agglutinin polypeptide. This nearly 300-fold increase in affinity results from combined enhancements of 15-to 20-fold each from the presence of an extended binding site for the oligosaccharide ligand and presentation of glycans on a protein, without any need to invoke multivalent binding.

### Effect of glycan branching on binding to a galactose-specific receptor

In order to demonstrate that the enhancement effects for individual C-type CRDs interacting with branched glycans presented on proteins represent a general phenomenon, it was of interest to examine another oligomeric receptor with substantially different organization and ligand-binding specificity. Oligomeric membrane-bound, glycan-binding receptors with C-type CRDs fall into two divergent groups on the basis of their primary binding specificity for mannose and related monosaccharides or galactose and related monosaccharides.^25^ While DC-SIGN falls in the mannose-binding group, the macrophage galactose lectin (MGL) is a representative galactose-binding receptor that in rat and humans consists of a single type of subunit,^26,27^ and is able to bind to viral glycoproteins, such as those on Ebola and other filoviruses.^28^ MGL is homologous to the hepatic asialoglycoprotein receptor and the sequence of the neck that links the CRDs to the membrane anchor is consistent with the presence of a potential coiled-coil domain that would stabilize an oligomeric structure like the trimer formed by the major subunit of the hepatic receptor.^29^ Chemical crosslinking and gel-filtration analysis confirmed that the extracellular domain of MGL expressed in bacteria forms trimers (Supplementary Data Fig. 1).

**Fig. 3:**
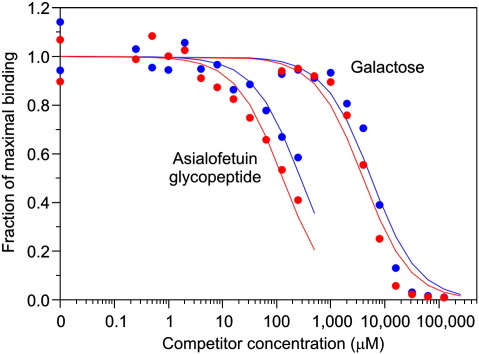
Binding competition assays comparing binding of a tri-antennary glycopeptide to MGL. Binding curves for monomeric CRD are shown in blue and curves for trimeric extracellular domain are shown in red. The *K*_I_ values obtained from these experiments are summarized in Table 2.

The effect of glycan branching on interaction with MGL was investigated by probing both monomeric CRDs and trimeric extracellular domains of MGL with a desialylated tri-antennary glycopeptide, isolated from fetuin, in the competition binding assay (Fig. 3). The relative affinity of the CRD for the glycopeptide compared to galactose is 21.5 (Table 1), which is 7.2-fold higher than the expected threefold enhancement that would be expected based solely on the presence of three terminal galactose residues. This roughly sevenfold enhanced affinity for branched glycans is somewhat like that observed for DC-SIGN (illustrated in Fig. 1b). Binding to the trimeric extracellular domain was enhanced a further 1.6-fold (Table 1), suggesting that binding of multiple terminal sugars by multiple CRDs in the extracellular domain (illustrated in Fig. 1f) makes only a small contribution to the overall affinity of the intact receptor for a branched glycan. The effect of branching on binding to MGL is consistent with previous results from testing this protein against a glycan array, because several of the ligands giving the highest signals for MGL are branched structures terminating in galactose,^30^ as well as quantitative binding assays with linear and branched sugar structures.^31,32^ However, the present results demonstrate that, as in the case of DC-SIGN, this enhancement results primarily from multiple interactions within a CRD and that only a limited further enhancement results from simultaneous interactions with multiple CRDs in a receptor oligomer.

### Effect of protein presentation on binding to MGL

**Fig. 4:**
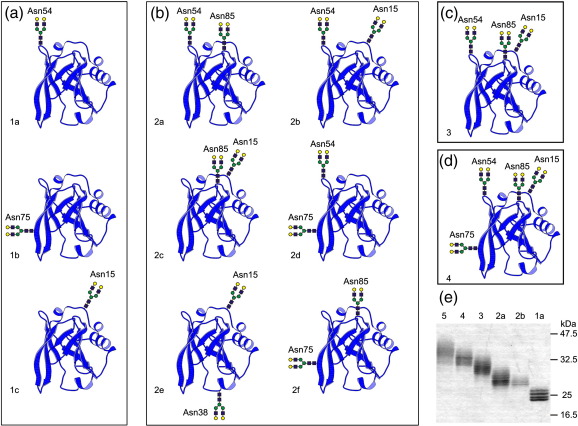
Creation and characterization of glycosylation variants of orosomucoid. (a)–(d) Modeled structures of mutants with 1 to 4 glycosylation sites. (e) SDS-PAGE of a selection of glycosylation variants. Glycoproteins purified by affinity chromatography on immobilized nickel columns were visualized on the gel by staining with Coomassie brilliant blue. The model was created with InsightII and Molscript^56^ based on the structure of bovine lactoglobulin (PDB ID 1B8E).^57^

To facilitate analysis of the additional factors that contribute to the affinity of MGL for glycoprotein ligands, variant forms of orosomucoid, a monomeric serum glycoprotein also known as α_1_-acid glycoprotein, that bear differing numbers of glycans were developed. Human orosomucoid contains five sites for N-linked glycosylation, each of which is occupied by a complex oligosaccharide (Fig. 4).^33^ Site-directed mutagenesis was used to generate vectors for the expression of modified forms of histidine-tagged orosomucoid with one to five glycosylation sites. The proteins were produced in Chinese hamster ovary cells, purified by affinity chromatography on chelated nickel columns, and treated with neuraminidase to remove terminal sialic acid residues. A selection of the desialylated glycoproteins separated by SDS-PAGE is shown in Fig. 4e. The differences in the mobilities of the proteins in the gel confirmed that selected glycosylation sites were missing, but that the remaining glycosylation sites are fully occupied. The multiple bands for each protein reflect the presence of different glycoforms of each variant. The glycans attached to the engineered forms of orosomucoid were characterized by single and tandem mass spectrometry (Supplementary Data Figs. 2 and 3) and compared in a semi-quantitative manner (Table 2).^34^ The results confirm that the sialic acid residues have been removed efficiently and indicate that each of the glycoproteins bears a mixture of bi-, tri-, and tetra-antennary complex glycans. Comparing the single-site variants reveals that the position of glycosylation does affect the degree of branching and this effect is carried over into the proteins with multiple glycosylation sites. However, there is no simple correlation between position or number of glycosylation sites and the extent of branching.

**Fig. 5:**
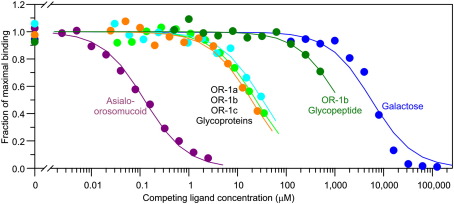
Competition experiments to quantify binding of orosomucoid variants to the CRD from MGL. Examples of ligands with a range of affinities are shown, with measured data shown as circles and fitted curves indicated as lines.

**Fig. 6:**
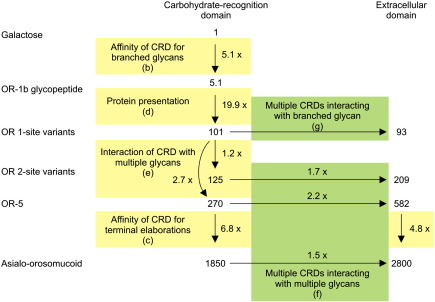
Summary of the sources of enhanced affinity for glycoproteins binding to MGL. The *K*_I_ values from Table 3 are linked by the fold enhancement resulting from various factors. Letters in parentheses refer to the illustrations in Fig. 1.

The glycoprotein presentation effect was investigated using the engineered glycoprotein ligands containing only a single glycosylation site as well as a glycopeptide pool from one of the variants. The results, illustrated in Fig. 5 and summarized in Table 3, reveal that the affinity of MGL for the three singly glycosylated variants of orosomucoid varies by a factor of less than 2, in spite of a range of nearly fivefold in the relative proportions of bi-and tri-antennary glycans attached to these variants. These results suggest that there is relatively little difference in the affinity of the receptor for the bi-and tri-antennary oligosaccharides. However, the relative affinity of the CRD of MGL for the glycopeptide from variant OR-1b (*K*_I,Gal_/*K*_I,glycopeptide_ =  12.4; Table 3) is roughly fivefold higher than the 2.3-fold enhancement that would be expected based on the presence of an average of 2.3 terminal galactose residues per glycopeptide. This number is almost as high as the sevenfold enhancement observed for the tri-antennary glycopeptide from asialofetuin (Table 1), confirming that the degree of branching makes only a modest contribution to enhanced affinity. The most striking comparison is the difference in affinities for the intact glycoproteins bearing a single glycan (*K*_I_ =  23 μM; Table 3) and the isolated glycopeptide derived from one of these glycoprotein (OR-1b, *K*_I_ =  450 μM; Table 3), which reveals a 20-fold enhancement in affinity resulting from presentation of the glycoprotein rather than the glycopeptide. Thus, for a glycoprotein bearing a single glycan interacting with a single CRD, there is a 100-fold enhancement in affinity even when normalized to the number of terminal galactose residues present, resulting from the fivefold effect of the affinity of the CRD for branched glycans and the 20-fold effect of presentation of the glycan on a protein. These contributions are summarized in Fig. 6.

The binding of the CRD of MGL to orosomucoid variants with increasing numbers of glycans reveals that the monomeric CRD has up to 2.7-fold increased affinity for orosomucoid variants with increasing numbers of glycans, as the *K*_I,Gal_/*K*_I,ligand_ value normalized to the number of terminal galactose residues increases from 101 to 270 on going from one to five glycans on the orosomucoid (Table 3). This increase could mean that, in addition to secondary interactions with branches within a glycan and with the protein to which the glycan is bound, the CRD can interact with other oligosaccharides attached to the glycoprotein ligand. However, it may also reflect the potential of some variants occasionally to bridge between two monomers immobilized on the polystyrene surface. Variation in the affinities of MGL for different variants of orosomucoid with two attached glycans probably reflects differences in the spacing of the glycans, but in the absence of more structural information about how the glycans project from the surface of the protein, it is difficult to discern a specific pattern correlating spacing with enhanced affinity.

### Effect of MGL oligomerization on binding to glycoprotein ligands

With knowledge of the effect of glycan branching and presentation on the affinity of individual CRDs for their ligands, it is possible to use the engineered glycoproteins to investigate the effect of receptor oligomerization on affinity for multivalent ligands. Binding of the singly glycosylated variants of orosomucoid to the trimeric extracellular domain of MGL does not differ significantly from binding to the monomeric CRD, indicating that binding of multiple CRDs in the trimer to branches of a single glycan on the glycoprotein is not a factor in enhanced glycoprotein ligand binding (Table 3 and Fig. 6). The 1.6-fold tighter binding of the free tri-antennary glycopeptide from asialofetuin to the trimer compared to the monomer (Table 1) might reflect accessibility not present in the protein-bound oligosaccharide.

Binding of multiple glycans on a target ligand to multiple CRDs in a receptor oligomer represents one form of affinity enhancement through multivalency that has been observed for soluble lectins (Fig. 1g). As expected for this effect, the affinity of the trimeric extracellular domain of MGL shows a further enhancement compared to the monomeric CRD when binding to orosomucoid variants with two or more glycans (Fig. 6). However, the magnitude of this enhancement is only about twofold, compared to much higher levels of enhancement observed for other glycan-binding proteins, such as pentameric bacterial toxins.^5^ The key difference in this case is that the natural targets for such toxins are membranes with multiple, widely spaced glycolipid ligands rather than simple soluble glycoproteins with relatively closely spaced glycans. Soluble multivalent ligands designed to interact with the toxins consist of multiple target glycans arrays on a scaffold that positions them at sufficient distances to interact with the multiple binding sites in the toxin oligomer.

### Further affinity enhancements in native asialo-orosomucoid

Comparison of the binding of MGL to asialo-orosomucoid derived from human blood with the protein bearing five glycans expressed in Chinese hamster ovary cells reveals that even with five glycans, the expressed protein has a lower affinity for both receptors than the natural protein (Table 3). As shown in Table 2, the orosomucoid from Chinese hamster ovary cells has a smaller fraction of tetra-antennary and a larger fraction of bi-antennary glycans compared to serum-derived asialo-orosomucoid,^33^ but the comparisons described earlier suggest that the increased degree of branching has only a modest effect on the affinity of binding to a CRD. Thus, it is likely that this difference reflects the presence of additional terminal glycan elaborations found on the natural protein but not synthesized in the cultured cells. For example, addition of outer arm fucose residues is known to be a common modification of serum orosomucoid,^35^ generating the Lewis^x^ epitope, which gives strong signals in glycan array analysis of MGL.^30^ Such structures are not found on the expressed proteins. A difference in binding of the natural and expressed proteins is observed for both the monomeric CRD and the trimeric extracellular domain, which is consistent with the suggestion that the difference lies in the interaction of individual CRDs with individual branching and terminal structures.

## Discussion

The observation that comparable affinity enhancements for binding of single protein-bound glycans to monomeric CRDs are observed for receptors as distantly related as DC-SIGN and MGL, and the fact that the enhancement is not unique to a glycan at a single site on a glycoprotein suggests a relatively nonspecific effect. At least two mechanisms can be suggested: low-specificity interactions between the surfaces of the CRD and the protein portion of the glycoprotein ligand, or restriction in the conformation of the glycans when they are attached to glycoprotein. As an illustration of the first mechanism, binding of the C-type CRD of E-selectin to P-selectin glycoprotein ligand 1 is enhanced by interaction with sulfated tyrosine residues in a region of the protein adjacent to the glycan that binds in the primary binding site of the CRD.^36^ Mutagenesis studies suggest that in this case, the negative charges on the sulfated tyrosine residues provide a relatively nonspecific interaction that adds to the overall affinity for the glycoprotein ligand. There is also substantial evidence that the flexibility of glycans is restricted when they are attached to proteins, which would lead to a decreased entropy penalty and hence increased affinity of a CRD for glycans presented in preferred conformations on glycoprotein backbones. Examples of glycoproteins in which the interaction of glycans with the protein surface have been examined in molecular detail include CD2,^37^ the α subunit of human chorionic gonadotropin,^38^ ribonuclease B,^39^ and viral envelope glycoproteins including the Epstein-Barr virus major envelope glycoprotein.^40^ Interactions between the glycan and protein portion of the glycoprotein typically occur between the first and second GlcNAc residues in the core and can result in the glycan lying parallel with the protein surface. Although the terminal residues of the glycan would remain accessible for lectin binding, the overall conformational space accessible to these glycans is substantially reduced.

The affinity measurements reported here were made in assay formats that allow us to dissect out effects associated with interactions of individual oligosaccharides and glycoproteins with individual CRDs and receptor oligomers. Combining the enhancements for binding of glycoproteins to MGL, as summarized in Table 3 and Fig. 6, results in an overall enhancement of nearly 3000-fold for binding of the natural, multiply glycosylated asialo-orosomucoid to the receptor trimer, corresponding to a sub-micromolar affinity. Multivalent interaction between glycoproteins and oligomeric receptors makes only a very modest contribution to this total enhancement.

When comparing the increase in affinity observed in these studies with enhancements of up to 10^6^-fold that have been reported for oligosaccharide binding to solubilized receptors or receptors on cell surfaces,^7,41^ and for soluble multivalent lectins such as the galectins,^42^ it is important to note that in the work presented here the interactions have been assessed in assays that segregate out the effects of two-and three-dimensional lattice formation (Fig. 1h and i). Three-dimensional lattice formation is well documented for the interactions of galectins with multivalent ligands,^7,8,43,44^ but such three-dimensional lattice formation, which probably contributes to high-affinity interaction in assays using solubilized membrane receptors, would not reflect a geometrically plausible arrangement for these receptors in their natural membrane environment. Two-dimensional lattice formation in membranes is a much more plausible explanation for the further enhancements observed in experiments on hepatocytes with natural or synthetic oligosaccharides or glycopeptides.^7,41,45^ Such enhancements have been observed with free oligosaccharides, corresponding to bridging of the type illustrated in the left-hand portion of Fig. 1h. However, the geometrical arrangement of binding sites in receptor oligomers and the extended interactions of the CRDs with oligosaccharide ligands might interfere with such interactions. In addition, free oligosaccharide or glycopeptide ligands have been observed in extended conformations when complexed with multivalent lectins in three-dimensional lattices.^43,46^ These structures suggest that the affinity for single glycans could be overestimated in such studies compared to what would be achieved for glycans that are attached to glycoproteins and are thus constrained in conformation. In the natural situation, two-dimensional lattice formation might be more likely to result from binding of multiple receptor oligomers to multiple glycans on a glycoprotein, as shown at the right in Fig. 1h. The possibility of pattern matching between fixed arrays of CRDs and the terminal sugars on oligosaccharides, either in receptor oligomers or in cell surface lattices has been suggested.^47^ However, experiments with DC-SIGN binding to oligosaccharides on a membrane surface indicate that, in some cases, receptor mobility and flexibility in the positioning of CRDs might be necessary for the receptors to adapt to the disposition of glycans on surfaces.^15^

## Material and Methods

### Expression of fragments from glycan-binding receptor

Expression of the extracellular domain and CRD from DC-SIGN^13^ and CRDs from rat MGL and RHL-1^12^ followed published procedures. The extracellular domain of DC-SIGN was further purified on a Mono-Q anion-exchange column.^13^ The extracellular domains of MGL (NCBI accession number P49301) from residue 59 to the C-terminus were expressed in an analogous T7 promoter system with an extra alanine residue appended at the N-terminus.

### Glycoproteins and glycopeptide preparation

Bovine fetuin (Sigma) and soybean agglutinin, prepared by affinity chromatography,^48^ were dissolved to 100 mg in 5 ml of 1% (w/v) ammonium bicarbonate and digested for 20 h at 37 °C with 5 mg of subtilisin (Sigma), resulting in short peptide fragments. Digested material was treated with 2 mM phenylmethylsulphonyl fluoride (Sigma; prepared at 30 mg/ml in ethanol) and lyophilized before being dissolved in 0.5 ml of 5% (v/v) acetic acid and loaded onto a Sephadex G-50 column (7 mm × 500 mm) run in 1% acetic acid.^49^ Sugar-containing fractions (0.5 ml) were pooled. Glycopeptides were treated with neuraminidase and repurified by passage through the Sephadex G-50 column.

### Production of orosomucoid variants

A cDNA for orosomucoid was cloned from a human liver cDNA library (ClonTech) using forward primer: AACCTCCTGGTCTCAGTATGGCGCTGTCCTGGG and reverse primer: TTCTAGTGATGGTGATGGTGATGTCCGGATTCCCCCTCCTCCTGTTTCCTCTC (Invitrogen) which created a His_6_ tag at the C-terminal end of the encoded protein. Glycosylation site mutations were created by inserting double-stranded synthetic oligonucleotides that included the desired mutations between appropriate restriction sites in the cDNA. In each case, the AAC or AAT codon for the asparagine residue of the N-glycosylation sequence (Asn-X-Ser/Thr) was mutated to the glutamine codon CAG. Mutations were verified by DNA sequencing, and the wild type and modified cDNAs were transferred into the eukaryotic expression vector pED.^50^ The dihydrofolate reductase-deficient Chinese hamster ovary (CHO) cell line DXB11 was transfected with mutant constructs using the calcium phosphate method.^51^ Following selection for two weeks in MEM alpha medium without nucleosides supplemented with 10% (v/v) dialyzed fetal calf serum, colonies were combined. Protein expression was amplified by passaging cells into medium containing methotrexate at 0.02 μM, 0.1 μM and 0.5 μM over a period of several weeks.

For production of protein, cells were grown to confluence in selection medium and transferred to serum-free medium: CHO-S-SFM II (Invitrogen) supplemented with 50 mM Hepes, pH 7.55, 4 μM CaCl_2_ and 0.5 μM methotrexate. Medium was harvested every two days for eight days, pooled, made to 0.5 M NaCl, 25 mM Tris–HCl, pH 7.8, 20 mM imidazole, centrifuged at 10,000 g for 15 min and loaded onto a 2 ml nickel-NTA-Sepharose column (Amersham Biosciences) equilibrated with N1 buffer (0.5 M NaCl, 25 mM Tris–HCl, pH 7.8). The column was washed with 8 ml of N1 buffer containing 20 mM imidazole and eluted with 8 × 1 ml of N1 buffer containing 100 mM imidazole. Fractions were analyzed by SDS-PAGE and those containing protein were pooled, diluted fivefold in N1 buffer and re-loaded onto a 2 ml Ni-NTA-Sepharose column. The column was eluted with 8 × 1 ml of N1 buffer containing 200 mM imidazole to concentrate the protein. The final protein-containing fractions were dialyzed against 50 mM sodium citrate, pH 6.0 and incubated with *Clostridium perfringens* neuraminidase (New England Biolabs) at 37 °C overnight. In order to remove the neuraminidase, fractions were made up to 0.5 M NaCl, 25 mM Tris–HCl, pH 7.8, 20 mM imidazole and loaded again onto a 2-ml Ni-NTA-Sepharose column and eluted with 8 × 1 ml of N1 buffer containing 200 mM imidazole. Protein yields were 1 ∼ 2 mg of desialylated variant orosomucoid per 100 ml of collected cell culture medium.

### Surface plasmon resonance studies

Analysis was done with a BiaCore 3000 instrument. CM5 sensor chips (Pharmacia Biosensor) were activated with *N*-hydroxysuccinimide and 1-ethyl-3-(3-dimethylaminopropyl) carbodiimide following the supplier's protocols. Coupling was performed for 10 min at a flow rate of 10 μl/min with 50 μg/ml ligand in 10 M sodium acetate, pH 5.0 for proteins and pH 4.0 for glycopeptide, followed by blocking with 1 M ethanolamine–HCl. All analysis was done in running buffer (10 mM Hepes, 150 mM NaCl, 5 mM CaCl_2_ with 0.005% (v/v) P20 (surfactant), pH 7.4), at 25 °C and a flow rate of 10 μl/min. Sensor chip surfaces were regenerated with EDTA-containing regeneration buffer. SigmaPlot was used to fit data to an equation for simple, saturable binding with a dissociation constant *K*_D_ superimposed on a linearly increasing background of non-specific binding.^12^

### Competition binding assays

Competition assays, in which receptor fragments were immobilized on polystyrene wells (Immulon 4 HBX from Thermo Labsystems), were done as described, using ^125^I-labeled Gal-BSA and ^125^I-labeled Man-BSA as a reporter ligands.^12,13^ In each experiment, duplicate titrations were performed and the average values for each concentration were used in a nonlinear, least-squares fitting program (SigmaPlot) to determine the concentrations of competing ligand required for 50% inhibition of reporter ligand binding (*K*_I_).^12^ The data are presented as average ± standard deviation for at least three independent experiments.

### Glycan analysis

Reduction and carboxymethylation were done as described.^52^ Samples were reduced at 37 °C for 1.5 h in 50 mM Tris–HCl buffer, pH 8.5, containing 2 mg/ml dithiothreitol, and carboxymethylated by reaction with 12 mg/ml iodoacetic acid at room temperature for 1 h. Carboxymethylation was terminated by dialysis against 50 mM ammonium bicarbonate, pH 8.5, at 4 °C for 36 h, followed by lyophilization. Samples were incubated with trypsin (Sigma) at a 50:1 (w/w) ratio in 50 mM ammonium bicarbonate, pH 8.5, for 16 h at 37 °C. The digestion was terminated by incubation at 100 °C for 3 min, followed by C18 Sep-Pak chromatography (Waters). Bound peptides were eluted with either 20% (v/v) or 40% (v/v) propanol in 5% (v/v) aqueous acetic acid, pooled and lyophilized. Digestion with peptide N-glycosidase F (Roche Molecular Biochemicals) was done in ammonium bicarbonate (50 mM, pH 8.5) for 16 h at 37 °C using 3 U of enzyme. The reaction was terminated by lyophilization and the released N-glycans were separated from peptides and O-glycopeptides by passage through a Sep-Pak C18 (Waters) column and permethylated using the NaOH procedure.^52^ Matrix-assisted laser desorption/ionization time-of-flight (MALDI-TOF) data were acquired on a Voyager-DE sSTR mass spectrometer (PerSeptive Biosystems) in the reflectron mode with delayed extraction. Permethylated samples were dissolved in 10 μl of 80% (v/v) methanol in water, and 1 μl of dissolved sample was premixed with 1 μl of matrix (10 mg/ml 2,5-dihydroxybenzoic acid in 80% (v/v) aqueous methanol) before loading onto a metal plate. The matrix-assisted laser desorption-ionization time-of-flight tandem mass spectrometry experiments were done with a 4800 Proteomics Analyzer (Applied Biosystems) operated in reflectron positive ion mode.

### Other analytical procedures

SDS-PAGE was done by the method of Laemmli.^53^ Sugar-containing fractions were assayed by the anthrone assay,^54^ and protein was assayed by the method of Bradford.^55^

## Figures and Tables

**Table 1:** Inhibition constants for asialofetuin glycopeptide binding to MGL

	CRD	ECD
*K*_I_ (μM)	260 ± 20	160 ± 45
*K*_Gal_/*K*_I_	21.5	35
Enhancement due to CRD affinity for branched glycans (fold)	7.2×	
Enhancement due to ECD affinity for branched glycans (fold)		11.7×
Enhancement due to multiple CRDs binding branches (fold)		1.6×

Binding inhibition experiments were done on plates coated with the CRD or ECD portions of MGL using ^125^I-labeled Gal-BSA as the reporter ligand.

**Table 2:** Distribution of N-linked glycans on variant asialo-orosomucoid molecules

Variant	Bi-antennary (%)	Tri-antennary (%)	Tetra-antennary (%)	Terminal galactose residues
1a	45	51	3	2.6
1b	67	30	2	2.3
1c	89	11	1	2.2
Average for single-site variants				2.4
2a	25	59	16	5.8
2b	47	47	5	5.1
2c	45	36	19	5.5
2d	32	53	16	5.8
2e	37	43	21	5.8
2f	40	40	20	5.6
Average for two-site variants				5.6
3	36	43	21	8.6
4	35	39	26	11.6
5	33	42	26	14.8
Serum-derived^a^	14	38	48	16.7

a Values from Ref. [33].

**Table 3:** Inhibition constants for binding to MGL

Ligand	CRD			ECD		
	*K*_I,ligand_ (μM)	*K*_I,Gal_/*K*_I,ligand_	*K*_I,Gal_/*K*_I,ligand_ per terminal Gal	*K*_I,ligand_ (μM)	*K*_I,Gal_/*K*_I,ligand_	*K*_I,Gal_/*K*_I,ligand_ per terminal Gal
Galactose	5600 ± 150					
OR-1b glycopeptide	450 ± 10	12.4	5.1			
OR-1a	31 ± 2			34 ± 1		
OR-1b	20 ± 6			20 ± 2		
OR-1c	17 ± 3			20 ± 1		
Average	23 ± 6	243	101	25 ± 7	224	93
OR-2a	4.7			3.1		
OR-2b	13			7.5		
OR-2c	11			2.1		
OR-2d	10			5.0		
OR-2e	2.3			8.5		
OR-2f	6.4			2.5		
Average	8 ± 4	700	125	4.8 ± 2.5	1170	209
OR-3	6.5	930	110	1.5	3700	434
OR-4	3.6	1550	134	0.9	6200	540
OR-5	1.4 ± 0.1	4000	270	0.65 ± 0.12	8615	582
Serum-derived OR	0.12 ± 0.01	31,000	1850	0.18 ± 0.02	47,000	2800

Binding inhibition experiments were done on plates coated with the CRD or ECD portions of MGL, using ^125^I-labeled Gal-BSA as the reporter ligand.
