# Supplementary material for: Identification of Novel Contributions to High-affinity Glycoprotein–Receptor Interactions using Engineered Ligands
Source: J Mol Biol. 2010 Feb 26;396(3):685–96. doi: 10.1016/j.jmb.2009.11.073 (PMC2824085; doi:10.1016/j.jmb.2009.11.073)
Supplement: Supplementary — material [file mmc1.pdf]

Supplementary Material (Not to be Published)
Click here to download Supplementary Material (Not to be Published): Supplementary material revised.pdf

Supplementary data for

Identification of novel contributions to high affinity glycoprotein-receptor interactions using engineered ligands

Peter J. Coombs, Rebecca Harrison, Samantha Pemberton, Adrián Quintero-Martinez, Simon Parry, Stuart M. Haslam, Anne Dell, Maureen E. Taylor, and Kurt Drickamer

Division of Molecular Biosciences, Department of Life Sciences, Imperial College, London SW7 2AZ, United Kingdom

Figures 1 - 3

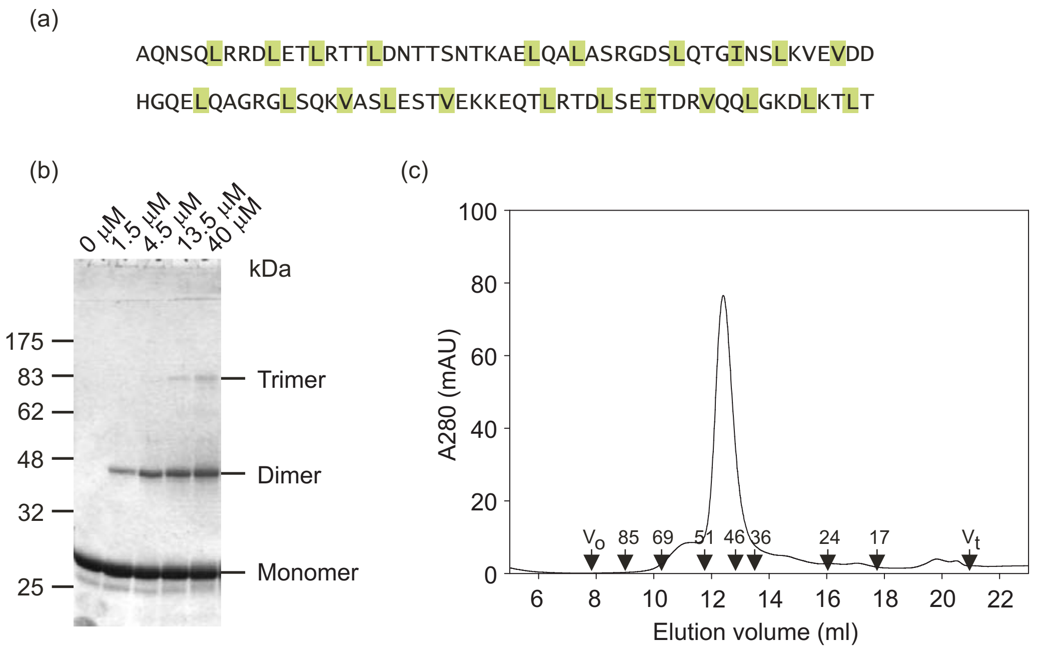

Supplementary Figure 1. Trimeric structure of the extracellular domain of MGL. (a) Potential coiled-coil domain in MGL. Positions *a* and *d* in the heptad repeats in the neck of MGL are indicated by shading. (b) Chemical crosslinking. Protein in 100 mM HEPES, pH 7.5 was reacted with the indicated concentrations of bis(sulfosuccinimidyl)suberate (Pearce Chemical Company) for 1 h at room temperature. The reaction was stopped by addition of 2x SDS-polyacrylamide gel sample buffer followed by heating and the products were resolved on a 10% gel that was stained with Coomassie blue. (c) Gel filtration analysis. Protein was run on a Superdex 200 column (1 cm × 30 cm; GE Healthcare) in 100 mM NaCl, 10 mM TrisHCl, pH 7.8, 2.5 mM EDTA at a flow rate of 0.5 ml/min. The positions of marker proteins are indicated by the Stokes radius: cytochrome c, 17 Å; bovine erythrocyte carbonic anhydrase, 23.9 Å; bovine serum albumin, 35.5 Å; yeast alcohol dehydrogenase, 45.5 Å; β-amylase, 51 Å; E. coli β-galactosidase, 69 Å; and thyroglobulin, 85 Å. The deduced Stokes radius of 47 Å corresponds closely to the value of 46-47 Å calculated using HYDRO[1], employing a bead model for a trimer based on the structure of the mannose-binding protein trimer (PDB ID: 1RTM) and extending the coiled-coil neck based on the sequence of MGL, which gives an overall length of ~200 Å for MGL.

Reference

1. Garcia de la Torre, J., Navarro, S., Lopez Martinez, M. C., Diaz, F. G. & Lopez Cascales, J. (1994). HYDRO: a computer software for the prediction of hydrodynamic properties of macromolecules. *Biophys. J.* **67**, 530–531.

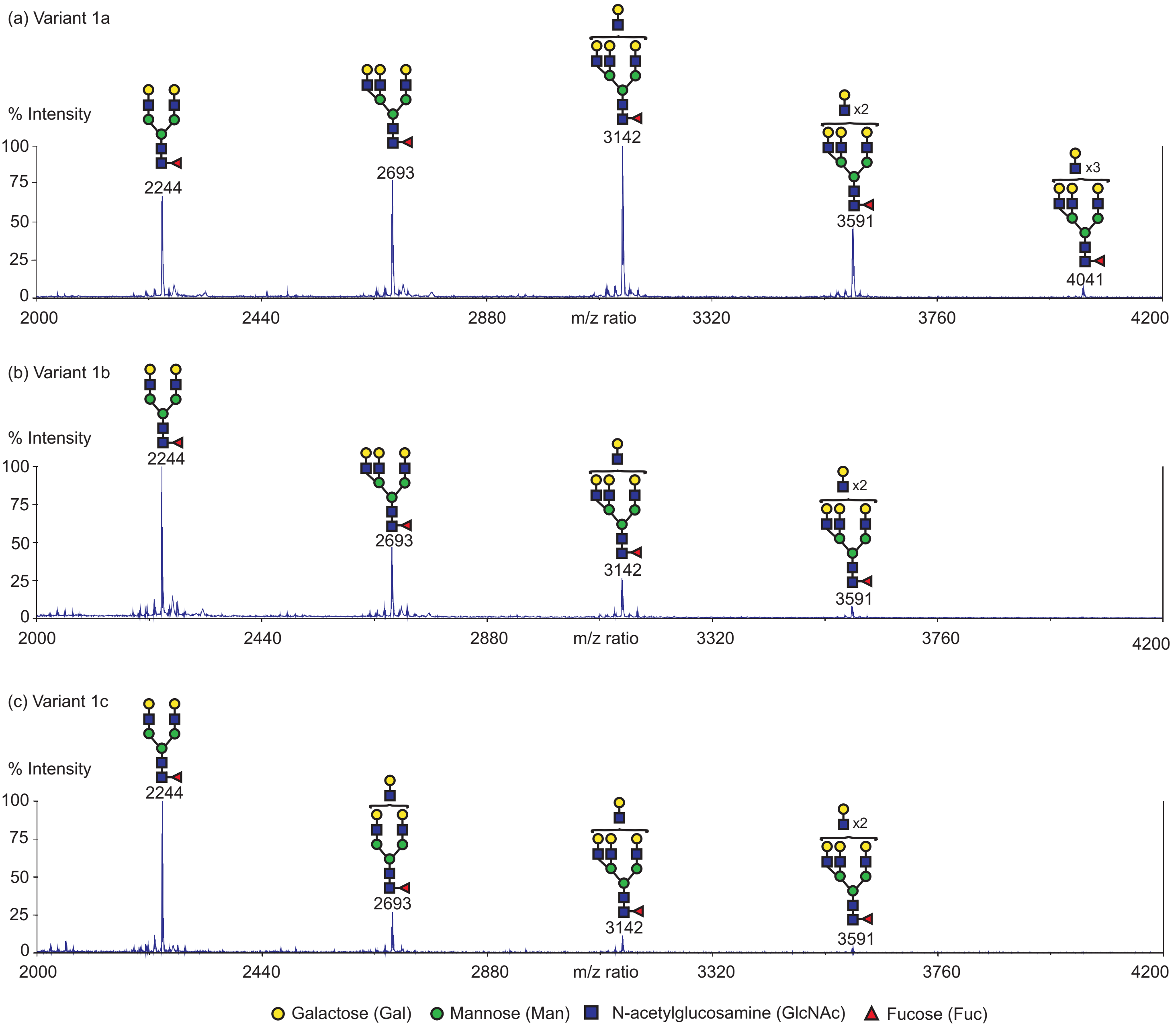

Supplementary Figure 2. MALDI-TOF mass spectra of N-linked glycans released from asialo-orosomucoid variants 1a, 1b and 1c. The N-linked glycans from the asialo-orosomucoid molecules were released by Peptide N-glycosidase F and permethylated. Data were acquired in the positive ion mode $[M+Na]^{+}$. Peak annotations are based on ^{12}C isotopic compositions, knowledge of the biosynthetic pathways and MS/MS analyses. The cartoon structures represent the most abundant structural isomer for each peak. Structures shown outside a bracket have not yet been unequivocally defined. For simplicity, only one potential branching pattern for tri-antennary structures is shown.

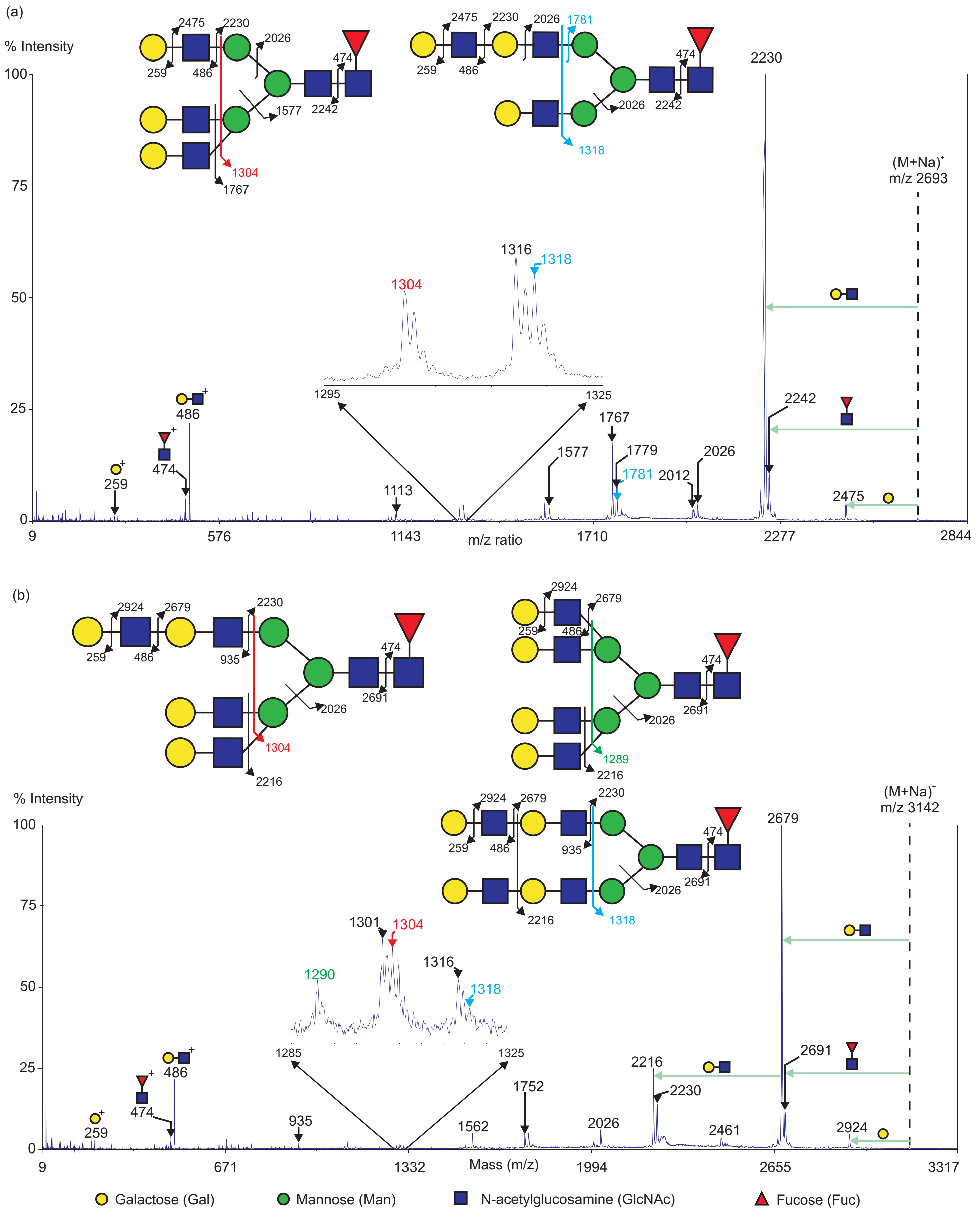

Supplementary Figure 3. Representative MALDI-TOF/TOF tandem mass spectra of variant asialo-orosomucoid N-glycans. (a) MS/MS spectrum of the molecular ion at m/z 2693. (b) MS/MS spectrum of the molecular ion at m/z 3142. The fragment ions are consistent with the sequences shown in the inset. The horizontal arrows on the spectra indicate losses from the molecular ion of the designated glycan moieties. Diagnostic fragment ions for bi-, tri-, and tetra-antennary structures are shown in blue, red, and green, respectively. Regions of diagnostic fragment ions have been expanded in order to deduce the relative abundances of the differently branched isomers.
